# Enhanced Olefin Transport by SiO_2_ Particles for Polymer/Ag Metal/Electron Acceptor Composite Membranes

**DOI:** 10.3390/polym12102316

**Published:** 2020-10-10

**Authors:** Sang Wook Kang

**Affiliations:** 1 Department of Chemistry, Sangmyung University, Seoul 03016, Korea; swkang@smu.ac.kr; Tel.: +82-2-2287-5362; 2 Department of Chemistry and Energy Engineering, Sangmyung University, Seoul 03016, Korea

**Keywords:** nanocomposite, silver metal, silica nanoparticle, olefin, facilitated transport

## Abstract

We showed the potential of poly(ethylene-*co*-propylene) (EPR)/silver metal/*p*-benzoquinone composite membranes for propylene/propane mixtures, i.e., a selectivity of 10 and a mixed gas permeance of 0.5 GPU (1 GPU = 1 × 10^−6^ cm^3^ (STP)/(cm^2^ s cmHg) in a previous study. In this study, we additionally found that the incorporation of fumed silica nanoparticles into EPR/silver metal/*p*-benzoquinone (*p*-BQ) composite membranes exhibited much higher permeance and selectivity for propylene/propane mixtures. The positive polarity of silver metal continuously increased with the increasing silica content up to the 0.1 weight ratio, as revealed by x-ray photoelectron spectroscopy (XPS). This increase in the polarity of silver metal was attributed to the enhanced interaction of *p*-BQ with the surface of Ag nanoparticles by the increased dispersion of *p*-BQ by fumed silica nanoparticles. Differential scanning calorimetry (DSC) also presented that the glass transition temperature (T_g_) of the membranes was almost invariant. Therefore, the improvement of the permeance and selectivity with the silica nanoparticles was attributable to the increased polarity of the silver metal rather than the structural change.

## 1. Introduction

During recent decades, there has been growing research interest in the fabrication of nanoparticles and nanocomposite materials due to their intriguing optical, electrical, and mechanical properties [1,2,3,4,5,6,7,8,9,10,11,12,13]. Among them, inorganic nanoparticles have been utilized in various applications, such as membranes, conducting materials, electrolytes, and nanocomposites [1,2,3,4,5,6,7,8,9,10,11,12,13]. In particular, the fumed silica nanoparticles were effectively used to enhance the permeability of poly(4-methyl-2-pentyne) (PMP) membranes by the increase of free volume for CO_2_ separation [2,4]. The incorporation of fumed silica nanoparticles caused the free volume to be increased, resulting in the enhancement of permeability. When micron-sized porous zeolite particles were introduced into organic polymer matrices, both the permeability and selectivity increased by the elasticity and processability with the zeolite pores. For the case of electrolytes, inorganic nanoparticles, such as zirconium(IV) oxide (ZrO_2_) were utilized for the enhancement of the conductivity of composites [14,15,16].

Recently, we reported on the novel application of silver nanoparticles as an olefin carrier for olefin/paraffin separation [17,18,19,20]. The electron acceptors, such as *p*-benzoquinone (*p*-BQ), 7,7,8,8-tetracyanoquinodimethane (TCNQ), and ionic liquids, such as 1-butyl-3-methyl imidazolium tetrafluoroborate (BMIIMBF_4_), could modify the surface energy on silver nanoparticles as the electron-insufficient state, complexing reversibly with olefin molecules [17,18,19,20]. Ag ions could be interacted with olefin molecules via reversible pi-complexation, and the propylene molecules could be rapidly transported onto the surface of particles for Ag nanoparticles as olefin carriers. For instance, the poly(ethylene-*co*-propylene) (EPR)/Ag nanoparticles/*p*-BQ composite membranes demonstrated a propylene/propane selectivity of 11 for mixture [17]. These membranes showed long-term stable separation performance for more than 100 h [17]. On the other hand, for composite membranes consisting of BMIMBF_4_ as ionic liquids and Ag nanoparticles, the selectivity and mixed-gas permeance were observed as 17 and 2.7 GPU, respectively [20]. These facilitated transport phenomena was generated by the positively polarized Ag nanoparticles through the interactions with counteranions in ionic liquids, resulting in enhancements in both the selectivity and mixed-gas permeance.

However, the separation performance of the nanocomposite membranes containing silver metal, such as nanoparticles, was relatively low, compared with that of polymer/silver salt complex membranes [17]. For example, while poly(2-ethyl-2-oxazoline) (POZ)/AgBF_4_ complex membranes showed a propylene/propane selectivity of 45 and a total mixed gas permeance of 12 GPU, the EPR/Ag nanoparticles/electron acceptor composite membranes showed the only 0.5 GPU (1 GPU = 1 × 10^−6^ cm^3^ (STP)/(cm^2^ s cmHg) [21]. These low mixed-gas permeances were attributable to the barrier effect of nanoparticles, resulting in the increase of transport path. However, since Ag ions were unstable as olefin carriers, the polymer/silver salt complex membranes showed poor stability with time. Therefore, polymer/silver metal composite membranes were seemingly more desirable for practical applications even though they have disadvantages, such as low permeance.

In this study, to enhance the separation performance of EPR/Ag nanoparticles/*p*-BQ composite membranes, we introduced fumed silica nanoparticles as an additive. We expected that the polarity of silver metal would be enhanced by the increased interaction of *p*-BQ with the surface of Ag nanoparticles by the dispersion of *p*-BQ by fumed silica nanoparticles. The detailed properties of permeance and selectivity for propylene/propane mixtures were examined to investigate the effect of silica nanoparticles. The membranes were further characterized using x-ray photoelectron spectroscopy (XPS) and differential scanning calorimetry (DSC).

## 2. Materials and Methods

### 2.1. Materials

Silver nanopowder (70 nm, 99.5%), *p*-benzoquinone, amorphous poly(ethylene-*co*-propylene) (EPR, M_w_ = 1.7 × 10^5^ g/mol, T_g_ = −50 °C, ethylene content 60 wt.%), and fumed silica nanoparticles (12-nm primary particles size) were purchased from Aldrich Chemical Co. All the chemicals were used as received.

### 2.2. Membrane Preparation and Permeance Measurements 

Polymer/silver nanoparticles/*p*-benzoquinone composite solutions were prepared by dissolving 0.1 g of Ag metal and 0.085 g of *p*-benzoquinone in 1.0 g of 10 wt.% EPR solution in toluene. Various weight ratios of fumed silica nanoparticles were incorporated into these composites. The composites containing fumed silica nanoparticles were coated on porous polymer support (polysulfone) by a coating machine (Control Coater RK Print-Coat instruments LTD, London, UK). The solvents in composites containing fumed silica nanoparticles were removed in an oven at room temperature, and then the composites were dried in a vacuum oven for 2 days. The flow rates for composite membranes were checked by mass flow controllers (MFC) and represented the permeance using a mass flow meter (MFM). The selectivity for the propylene/propane mixture (50:50 vol%) was measured by gas chromatography (Hewlett Packard, Palo Alto, CA, USA) with a thermal conductivity detector (TCD) and operated at 20 °C and 40 psig. 

### 2.3. Characterization 

X-ray photoelectron spectroscopy (XPS) was performed using an X-ray photoelectron spectrometer with Mg X-ray source at 300 W (Waltham, MA, USA). The glass transition temperatures (GPC) was measured using a Perkin-Elmer DSC-7 (Waltham, MA, USA) at the heating rate of 20 °C/min. The IR measurements were performed on a Nicolet FT-IR spectrometer (Waltham, MA, USA). For these measurements, 32 scans were signal-averaged at a resolution of 4 cm^−1^. The spectroscopic characterization was performed using a pressure cell equipped with KBr windows.

## 3. Results and Discussion

### 3.1. Coated State of Composite Membranes

**Figure 1:**
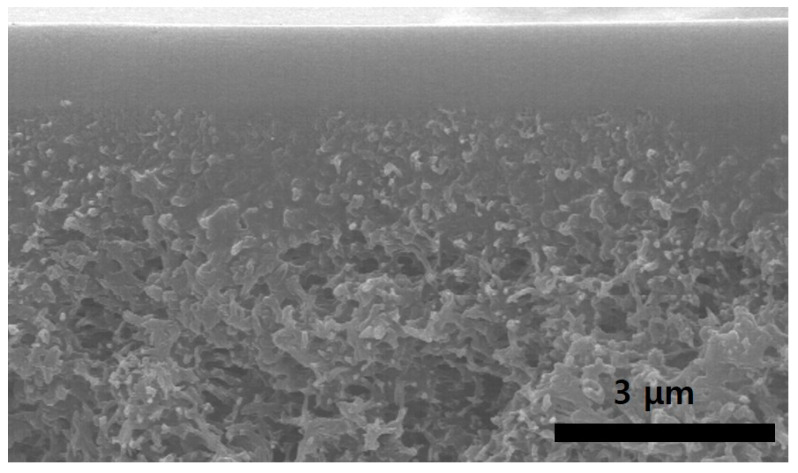
SEM image of poly(ethylene-*co*-propylene) (EPR)/Ag metal/*p*-benzoquinone (*p*-BQ)/SiO_2_ composite membranes coated onto polysulfone.

Figure 1 shows the SEM image of a 1/1/0.85 EPR/Ag metal/*p*-BQ/SiO_2_ composite membrane coated onto polysulfone. As shown in Figure 1, the selective layer consisting of the EPR/Ag metal/*p*-BQ/SiO_2_ composite was well coated onto the porous polymer support. In particular, the phase separation phenomena were not observed by the introduction of SiO_2_, indicating that the silica particles were well dispersed and stabilized in the EPR chains.

### 3.2. Separation Performance: Permeance and Selectivity

**Figure 2:**
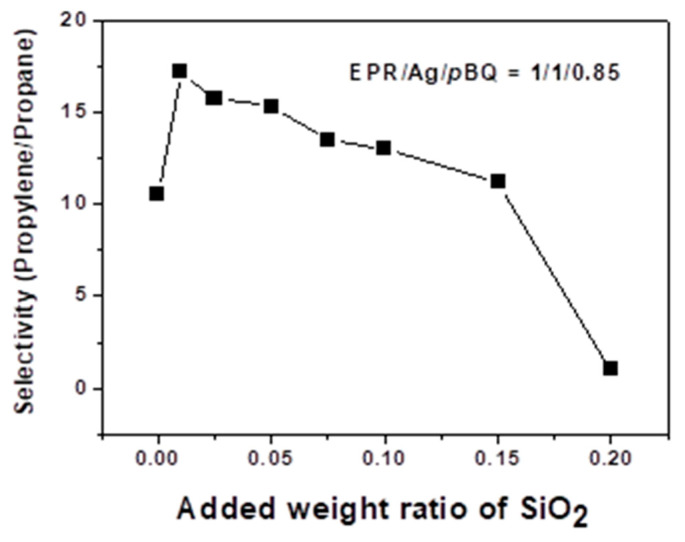
Separation performance: mixed gas selectivity of EPR/Ag metal/*p*-BQ membranes with various weight ratios of fumed silica nanoparticles.

**Figure 3:**
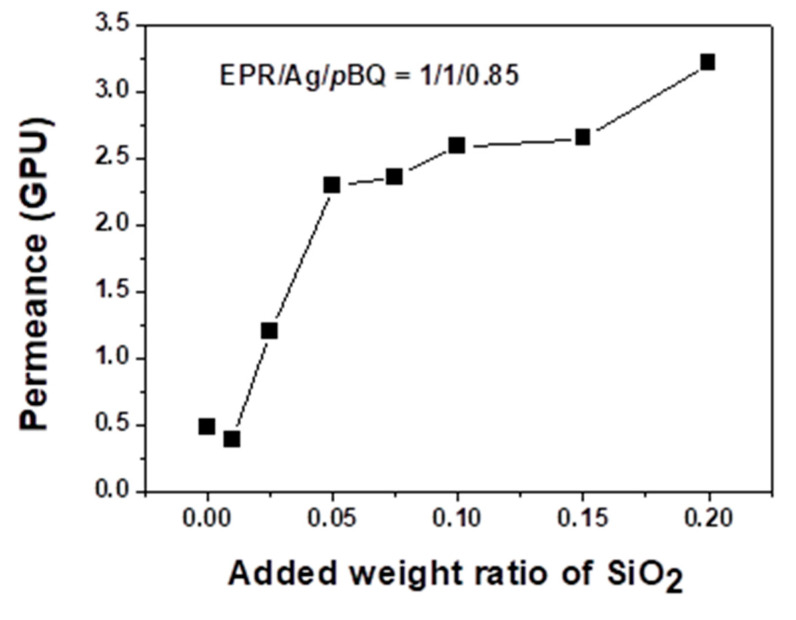
Separation performance: mixed gas permeance of EPR/Ag metal/*p*-BQ membranes with various weight ratios of fumed silica nanoparticles.

Figure 2 and Figure 3 show the total mixed-gas permeance and selectivity of the propylene/propane mixture through EPR/Ag nanoparticle/*p*-BQ composite membranes containing the fumed silica nanoparticles. The weight ratio of EPR, silver metal and *p*-BQ was fixed at 1:1:0.85. The separation of propylene/propane mixtures using 1:1:0.85 EPR:Ag nanoparticles:*p*-BQ nanocomposite was evaluated for various concentrations of fumed silica nanoparticles. The composition ratio of 1:1:0.85 EPR:Ag nanoparticles:*p*-BQ was selected as the best separation performance was observed for this ratio in a previous study [21]. As the *p*-BQ contents increased above the 0.85 ratio, phase separation was observed, resulting in the decrease of the separation performance.

The EPR/Ag nanoparticle/*p*-BQ composite membranes without silica nanoparticles exhibited low gas permeation and selectivity of the propylene/propane mixtures, the mixed gas permeance was ca. 0.5 GPU, and the selectivity of propylene/propane was 11. The experiments were performed three times, and average values were used (error range = ±1). When the fumed silica nanoparticles were introduced into the EPR/Ag nanoparticle/*p*-BQ composite, both the propylene and propane permeance slightly decreased up to the 0.01 weight ratio, possibly explained by the Maxwell relation. As the pathway of gas molecules increased in polymer chains with the presence of SiO_2_ particles, the gas permeance would be diminished. Thus, we predicted that, at small amounts of fumed silica nanoparticles, the silica nanoparticles played a role as barriers for gas transport. However, as the concentration of fumed silica nanoparticles increased up to the 0.15 weight ratio, the facilitated olefin transport was enhanced. 

In particular, the presence of the fumed silica nanoparticles in the EPR/Ag metal/*p*-benzoquinone composite membranes resulted in an increase in both the permeance and selectivity for the propylene/propane mixture. The selectivity of propylene/propane and the mixed gas permeance increased to 17 and 2.5 GPU, respectively at the 0.01 weight ratio of fumed silica nanoparticles. However, above the 0.15 weight ratio of fumed silica nanoparticles, the membranes resulted in a significant increase of permeance to give essentially no selectivity of propylene/propane mixtures, which was due to the interfacial defects by the aggregation of silica nanoparticles with EPR polymer chains. The generated interfacial defects between the polymer chains and the fumed silica nanoparticles resulted in a decrease in selectivity.

The increase of propylene permeance was attributable to the increase of facilitated olefin transport by the incorporation of fumed silica nanoparticles. We propose that the presence of fumed silica nanoparticles caused the *p*-BQ to be well dispersed in polymer matrix, resulting in the interactions between *p*-BQ and the surface of Ag nanoparticles being enhanced, which was characterized by XPS. On the other hand, above the 0.15 weight ratio, both the propylene and propane permeance reached 3.3 GPU, due to the interfacial defects of membranes by the aggregation of silica nanoparticles. The generated interfacial defects caused both propylene and propane molecules to be rapidly transported, diminishing the selectivity.

### 3.3. Binding Energy and FT-IR Spectra

**Figure 4:**
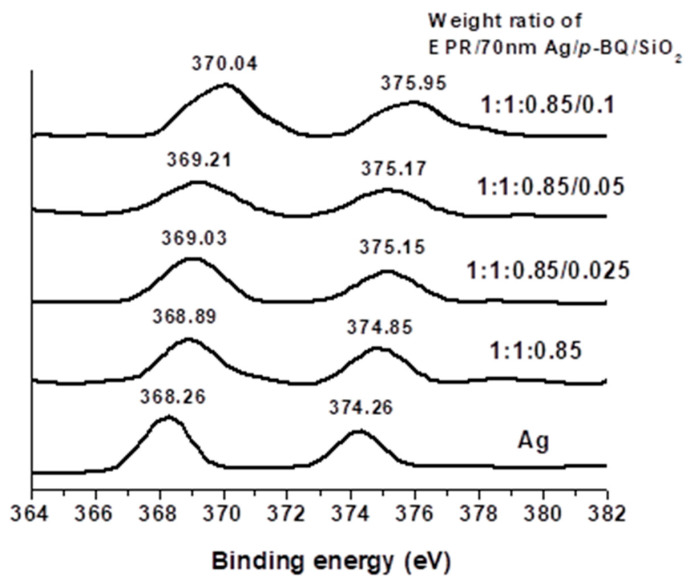
Binding energies for the silver metal in EPR/Ag nanoparticles/*p*-BQ with varying contents of silica nanoparticles.

XPS was used to observe the change of the chemical environment around the silver nanoparticles in EPR/Ag nanoparticles/*p*-BQ composite membranes upon the incorporation of silica nanoparticles. As the d_5/2_ orbital of Ag was measured at the value of 368.26 eV, the change of electron density for Ag nanoparticles was observed at these ranges [14]. When XPS was analyzed, C 1s at 285.0 eV was utilized as the standard value. The binding energy of the d_5/2_ orbital of the silver particle in the EPR/Ag nanoparticle/*p*-BQ composites increased gradually from 368.89 to 370.04 eV with the increasing silica nanoparticle content as shown in Figure 4. This indicated that the binding energy of the valence electrons in the silver atoms increased due to the incorporation of fumed silica nanoparticles. We propose that the incorporation of fumed silica nanoparticles caused the surface of silver nanoparticles to be more positively polarized by the increased interactions with the *p*-BQ dispersed in the polymer matrix.

**Figure 5:**
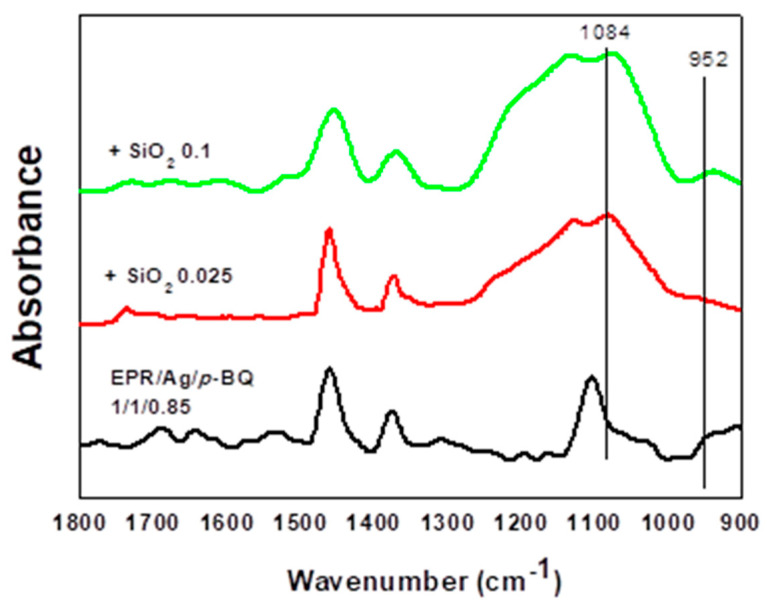
FT-IR spectra of 1/1/0.85EPR/Ag nanoparticles/*p*-BQ composites with varying contents of silica nanoparticles.

To analyze the change of strength in bonding, FT-IR spectroscopy was investigated as shown in Figure 5. The signals at 1079–1088 cm^−1^ and 952–953 cm^−1^ were attributed to asymmetric vibrations of Si–O–Si and asymmetric vibrations of Si-OH, respectively. As the weight ratio of fumed silica nanoparticles increased, the peak at 952–953 cm^−1^ was shifted to a lower wavenumber, indicating that the bond-strength in Si–OH became weakened due to the interaction with *p*-BQ. These interactions caused *p*-BQ to be well dispersed in the polymer matrix, resulting in the favorable interactions with the surface of Ag nanoparticles. Thus, the coordinative interactions between *p*-BQ and silica nanoparticles as additives were confirmed by FT-IR spectra.

### 3.4. Glass Transition Temperature

**Figure 6:**
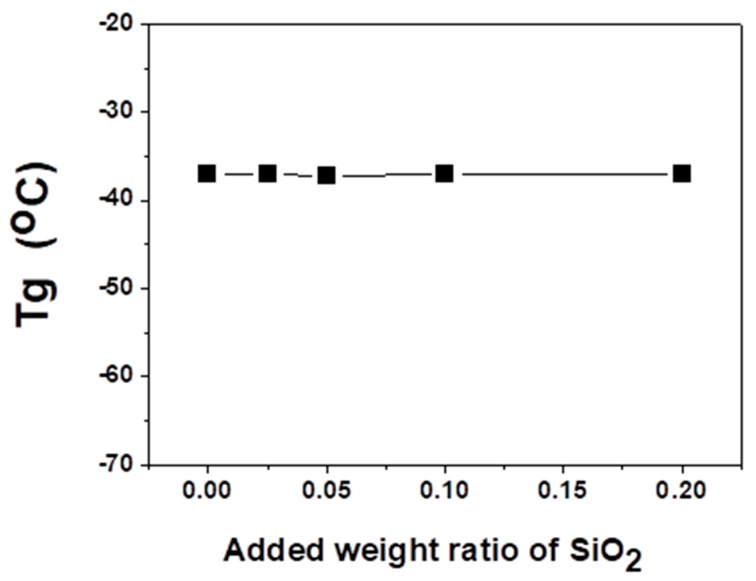
The glass transition temperature (T_g_) of 1/1/0.85 EPR/Ag metal/*p*-benzoquinone composites with varying contents of silica nanoparticles.

The change in chain mobility of polymer composite membranes containing the fumed silica nanoparticles was observed by measuring the glass transition temperature (T_g_). Figure 6 shows that T_g_ was found to be −37 °C for 1/1/0.85 EPR/Ag nanoparticles/*p*-BQ, but it remained constant at −37 °C within the error range with increasing silica concentration up to the 0.2 weight ratio of silica nanoparticles. These results represented that the effect of fumed silica nanoparticles on both the chain flexibility and the structure property of composite membranes was negligible Thus, we concluded that the increased propylene permeance was not attributable to the increased chain flexibility but to the increased polarity of silver nanoparticles from the introduction of silica nanoparticles. Similar results, such as the decrease in the separation performance due to interfacial defects, were also observed in previous studies [17,18].

## 4. Conclusions

In this study, we investigated the effect of silica nanoparticles on the separation performance through EPR/Ag nanoparticle/*p*-BQ composite membranes. When fumed silica nanoparticles were introduced into the 1:1:0.85 EPR/Ag nanoparticle/*p*-BQ composite membrane, the permeance and the selectivity for the propylene/propane mixture were improved simultaneously. The increased propylene permeance was not attributable to the increased chain flexibility but to the increased polarity of silver nanoparticles by the introduction of fumed silica nanoparticles, as confirmed by DSC and XPS analysis. The increased positive polarity of silver nanoparticles helped to react with olefin molecules more favorably, resulting in the enhancement of facilitated olefin transport. These results could be explained by the fact that the presence of fumed silica nanoparticles caused the *p*-BQ to be well dispersed in polymer matrix, resulting in the interactions between *p*-BQ and the surface of the Ag nanoparticles becoming enhanced. Thus, the facilitated olefin transport was accelerated and as a result, both the selectivity and mixed-gas permeance were enhanced.
